# Supplementary material for: Amyloid and tau accumulate across distinct spatial networks and are differentially associated with brain connectivity
Source: eLife. 2019 Dec 9;8:e50830. doi: 10.7554/eLife.50830 (PMC6938400; doi:10.7554/eLife.50830)
Supplement: Supplementary file 1. [file elife-50830-supp1.docx]

**Supplementary Table 1. Association between functional MRI network signals with the amyloid-β and tau networks SUVRs**

|  | **Resting-state functional MRI networks** | | | | | | | | | | | | | | | | |
| --- | --- | --- | --- | --- | --- | --- | --- | --- | --- | --- | --- | --- | --- | --- | --- | --- | --- |
|  | **Primary Visual** | **Secondary Visual** | | **Extra-striate Visual** | | **Anterior Cingulate** | **Posterior DMN** | | **Left**  **Fronto-Parietal** | | **Dorsal Attention** | | **Limbic** | | **Right Fronto-Parietal** | | **Basal Ganglia** |
| amyloid-β IC 2  amyloid-β IC 3  amyloid-β IC 4  amyloid-β IC 5  amyloid-β IC 6  amyloid-β IC 7  amyloid-β IC 8  amyloid-β IC10  amyloid-β IC 11  tau IC 2  tau IC 3  tau IC 4  tau IC 5  tau IC 6  tau IC 7  tau IC 8  tau IC 10  tau IC 11 | -0.030  (0.814)  -0.173  (0.174)  -0.084  (0.515)  -0.070  (0.587)  -0.173  (0.175)  -0.101  (0.431)  -0.043  (0.740)  -0.158  (0.216)  -0.082  (0.525)  -0.179  (0.160)  -0.091  (0.478)  -0.178  (0.162)  -0.218  (0.086)  -0.275  (0.029)  -0.197  (0.123)  -0.055  (0.667)  -0.186  (0.144)  -0.254  (0.045) | 0.032  (0.806)  -0.106  (0.407)  -0.085  (0.506)  -0.032  (0.805)  -0.126  (0.325)  -0.061  (0.636)  0.023  (0.861)  -0.112  (0.381)  -0.043  (0.740)  -0.136  (0.288)  -0.019  (0.882)  -0.137  (0.283)  -0.183  (0.152)  -0.218  (0.087)  -0.144  (0.260)  0.022  (0.866)  -0.129  (0.313)  -0.189  (0.138) | | -0.054  (0.677)  -0.224  (0.078)  -0.124  (0.335)  -0.166  (0.195)  -0.214  (0.092)  -0.123  (0.339)  -0.089  (0.489)  -0.228  (0.072)  -0.119  (0.352)  -0.157  (0.220)  -0.092  (0.473)  -0.195  (0.126)  -0.223  (0.079)  -0.281  (0.026)  -0.184  (0.149)  -0.061  (0.633)  -0.203  (0.111)  -0.258  (0.042) | | 0.063  (0.625)  -0.117  (0.363)  -0.021  (0.869)  -0.037  (0.773)  -0.081  (0.528)  -0.113  (0.379)  -0.043  (0.740)  -0.095  (0.460)  0.041  (0.748)  -0.062  (0.631)  -0.171  (0.182)  -0.059  (0.649)  -0.039  (0.765)  -0.089  (0.490)  -0.070  (0.584)  -0.116  (0.366)  -0.052  (0.684)  -0.030  (0.818) | -0.069  (0.592)  -0.191  (0.133)  -0.082  (0.526)  -0.132  (0.301)  -0.148  (0.246)  -0.122  (0.341)  -0.060  (0.640)  -0.189  (0.138)  -0.112  (0.382)  -0.158  (0.216)  -0.096  (0.453)  -0.144  (0.259)  -0.177  (0.166)  -0.226  (0.075)  -0.150  (0.242)  -0.073  (0.568)  -0.162  (0.204)  -0.219  (0.085) | | -0.016  (0.899)  -0.201  (0.114)  -0.055  (0.666)  -0.100  (0.438)  -0.178  (0.164)  -0.066  (0.608)  -0.049  (0.704)  -0.212  (0.095)  -0.024  (0.852)  -0.143  (0.264)  -0.108  (0.399)  -0.131  (0.306)  -0.119  (0.352)  -0.221  (0.082)  -0.118  (0.357)  -0.071  (0.579)  -0.173  (0.175)  -0.166  (0.194) | | -0.086  (0.503)  -0.219  (0.085)  -0.093  (0.468)  -0.172  (0.178)  -0.202  (0.113)  -0.128  (0.318)  -0.111  (0.385)  -0.251  (0.047)  -0.066  (0.607)  -0.160  (0.212)  -0.114  (0.375)  -0.158  (0.216)  -0.150  (0.242)  -0.234  (0.065)  -0.129  (0.312)  -0.102  (0.425)  -0.189  (0.139)  -0.186  (0.145) | | 0.049  (0.702)  -0.091  (0.479)  -0.029  (0.824)  -0.076  (0.555)  -0.057  (0.659)  -0.105  (0.412)  -0.054  (0.677)  -0.092  (0.472)  -0.055  (0.668)  -0.113  (0.378)  -0.086  (0.504)  -0.096  (0.453)  -0.094  (0.463)  -0.189  (0.137)  -0.100  (0.435)  -0.106  (0.410)  -0.094  (0.464)  -0.141  (0.269) | | -0.015  (0.906)  -0.204  (0.109)  -0.036  (0.781)  -0.086  (0.504)  -0.197  (0.122)  -0.080  (0.531)  -0.070  (0.586)  -0.215  (0.091)  0.020  (0.877)  -0.086  (0.502)  -0.068  (0.598)  -0.099  (0.442)  -0.062  (0.630)  -0.201  (0.115)  -0.065  (0.612)  -0.034  (0.790)  -0.125  (0.330)  -0.117  (0.361) | | 0.078  (0.545)  0.015  (0.908)  0.093  (0.470)  0.109  (0.395)  0.037  (0.775)  -0.075  (0.558)  0.089  (0.488)  0.047  (0.717)  -0.016  (0.900)  -0.092  (0.474)  <0.001  (0.995)  0.027  (0.834)  -0.009  (0.945)  -0.046  (0.723)  -0.027  (0.832)  -0.004  (0.977)  0.032  (0.807)  -0.037  (0.774) |
|  | **Resting-state functional MRI networks (continuation)** | | | | | | | | | | | | | | | | |
|  | **Anterior DMN** | | **Medial Executive Control** | | **Sensorimotor** | | | **Posterior Salience** | | **Anterior Salience** | | **Language** | | **Somato-sensory** | | **Lateral Executive Control** | |
| amyloid-β IC 2  amyloid-β IC 3  amyloid-β IC 4  amyloid-β IC 5  amyloid-β IC 6  amyloid-β IC 7  amyloid-β IC 8  amyloid-β IC 10  amyloid-β IC 11  tau IC 2  tau IC 3  tau IC 4  tau IC 5  tau IC 6  tau IC 7  tau IC 8  tau IC 10  tau IC 11 | 0.044  (0.730)  -0.135  (0.291)  0.012  (0.926)  -0.008  (0.950)  -0.144  (0.262)  -0.079  (0.538)  -0.016  (0.902)  -0.149  (0.244)  0.053  (0.680)  -0.053  (0.682)  -0.045  (0.728)  -0.036  (0.780)  -0.010  (0.940)  -0.131  (0.305)  -0.027  (0.833)  -0.013  (0.920)  -0.067  (0.604)  -0.040  (0.754) | | -0.046  (0.722)  -0.163  (0.203)  -0.061  (0.634)  -0.104  (0.419)  -0.128  (0.319)  -0.124  (0.335)  -0.067  (0.603)  -0.152  (0.233)  -0.094  (0.462)  -0.175  (0.169)  -0.111  (0.388)  -0.167  (0.190)  -0.164  (0.200)  -0.244  (0.054)  -0.167  (0.192)  -0.093  (0.467)  -0.182  (0.155)  -0.209  (0.100) | | -0.136  (0.289)  -0.284  (0.024)  -0.165  (0.196)  -0.190  (0.136)  -0.285  (0.024)  -0.155  (0.226)  -0.168  (0.190)  -0.296  (0.018)  -0.059  (0.644)  -0.187  (0.142)  -0.157  (0.219)  -0.148  (0.246)  -0.117  (0.360)  -0.207  (0.103)  -0.140  (0.275)  -0.086  (0.502)  -0.177  (0.166)  -0.143  (0.265) | | | 0.056  (0.663)  -0.028  (0.827)  0.087  (0.497)  0.047  (0.715)  -0.043  (0.738)  -0.137  (0.284)  0.022  (0.862)  -0.047  (0.713)  -0.012  (0.925)  -0.045  (0.727)  0.032  (0.803)  -0.004  (0.974)  0.033  (0.795)  -0.071  (0.579)  0.006  (0.961)  0.002  (0.989)  -0.013  (0.917)  -0.011  (0.932) | | 0.014  (0.911)  -0.134  (0.296)  0.023  (0.857)  -0.015  (0.907)  -0.139  (0.276)  -0.095  (0.457)  -0.026  (0.837)  -0.140  (0.275)  0.026  (0.841)  -0.098  (0.446)  -0.050  (0.699)  -0.065  (0.612)  -0.020  (0.876)  -0.144  (0.259)  -0.056  (0.662)  -0.023  (0.857)  -0.088  (0.492)  -0.059  (0.648) | | -0.018  (0.889)  -0.097  (0.448)  -0.002  (0.990)  -0.077  (0.548)  -0.077  (0.547)  -0.108  (0.400)  -0.041  (0.750)  -0.116  (0.367)  -0.077  (0.546)  -0.163  (0.201)  -0.076  (0.555)  -0.145  (0.258)  -0.128  (0.318)  -0.208  (0.102)  -0.125  (0.327)  -0.084  (0.515)  -0.162  (0.206)  -0.165  (0.195) | | -0.014  (0.915)  -0.129  (0.314)  0.018  (0.889)  -0.062  (0.627)  -0.128  (0.318)  -0.126  (0.327)  -0.050  (0.695)  -0.159  (0.214)  -0.026  (0.842)  -0.074  (0.564)  -0.032  (0.806)  -0.092  (0.474)  -0.058  (0.650)  -0.167  (0.192)  -0.050  (0.697)  -0.054  (0.676)  -0.114  (0.373)  -0.090  (0.481) | | -0.021  (0.869)  -0.151  (0.238)  -0.026  (0.839)  -0.071  (0.579)  -0.137  (0.283)  -0.134  (0.297)  -0.060  (0.638)  -0.158  (0.217)  -0.034  (0.792)  -0.087  (0.500)  -0.056  (0.664)  -0.076  (0.553)  -0.051  (0.691)  -0.161  (0.209)  -0.079  (0.539)  -0.041  (0.749)  -0.107  (0.403)  -0.100  (0.438) | |

The values presented in the table correspond to Spearman’s Rho followed by (p values) for the correlations between functional MRI and PET networks, while controlling for age, sex and presence of cognitive impairment. Underlined values correspond to significant group differences without adjusting for multiple comparisons. Correlations were carried out in all amyloid positive individuals. DMN, default-mode network. Amyloid-β and tau independent components (IC) can be found in Figure 1.
