# Supplementary material for: Amyloid and tau accumulate across distinct spatial networks and are differentially associated with brain connectivity
Source: eLife. 2019 Dec 9;8:e50830. doi: 10.7554/eLife.50830 (PMC6938400; doi:10.7554/eLife.50830)
Supplement: Supplementary file 2. [file elife-50830-supp2.docx]

**Supplementary Table 2. Association between gray matter volumes in vulnerable regions to Alzheimer’s disease with the amyloid-β and tau networks SUVRs**

|  | **amyloid-β IC 2** | **amyloid-β IC 3** | **amyloid-β IC 4** | **amyloid-β IC 5** | **amyloid-β IC 6** | **amyloid-β IC 7** | **amyloid-β IC 8** | **amyloid-β IC 10** | **amyloid-β IC 11** |
| --- | --- | --- | --- | --- | --- | --- | --- | --- | --- |
| Gray matter volumes | -0.0176 (0.872) | -0.0762 (0.483) | -0.0998 (0.358) | -0.0817 (0.452) | -0.1581 (0.144) | **-0.2523 (0.018)** | -0.1140 (0.293) | -0.1164 (0.283) | -0.0939 (0.387) |
|  | **tau**  **IC 2** | **tau**  **IC 3** | **tau**  **IC 4** | **tau**  **IC 5** | **tau**  **IC 6** | **tau**  **IC 7** | **tau**  **IC 8** | **tau**  **IC 10** | **tau**  **IC 11** |
| Gray matter volumes | **-0.3321 (0.002)** | **-0.3545 (<0.001)** | **-0.2995 (0.005)** | **-0.2573 (0.016)** | **-0.3471 (<0.001)** | **-0.4193 (<0.001)** | **-0.3233 (0.002)** | **-0.3824 (<0.001)** | **-0.3576 (<0.001)** |

The values presented in the table correspond to Spearman’s Rho followed by (p values) for the correlations between the gray matter volumes of regions that show atrophy in Alzheimer’s disease (Darby et al., 2019) and PET networks SUVRs, while controlling for age, sex, presence of cognitive impairment and intracranial volume. P values in bold correspond to significant group differences after adjusting for multiple comparisons with false discovery rate corrections (FDR) (q < 0.05).
