# Supplementary material for: Amyloid and tau accumulate across distinct spatial networks and are differentially associated with brain connectivity
Source: eLife. 2019 Dec 9;8:e50830. doi: 10.7554/eLife.50830 (PMC6938400; doi:10.7554/eLife.50830)
Supplement: Transparent reporting form [file elife-50830-transrepform.pdf]

eLIFE
1st Floor
24 Hills Road
Cambridge CB2 1JP, UK
P 01223 855340
W elifesciences.org
T @elife

eLife's transparent reporting form

We encourage authors to provide detailed information *within their submission* to facilitate the interpretation and replication of experiments. Authors can upload supporting documentation to indicate the use of appropriate reporting guidelines for health-related research (see EQUATOR Network), life science research (see the BioSharing Information Resource), or the ARRIVE guidelines for reporting work involving animal research. Where applicable, authors should refer to any relevant reporting standards documents in this form.

If you have any questions, please consult our Journal Policies and/or contact us: editorial@elifesciences.org.

Sample-size estimation

- You should state whether an appropriate sample size was computed when the study was being designed
- You should state the statistical method of sample size computation and any required assumptions
- If no explicit power analysis was used, you should describe how you decided what sample (replicate) size (number) to use

Please outline where this information can be found within the submission (e.g., sections or figure legends), or explain why this information doesn't apply to your submission:

We included all the subjects from the Swedish Biofinder Study (http://biofinder.se/) with available 18F-Flortaucipir PET, 18F-Flutemetamol PET and structural MRI data.

Replicates

- You should report how often each experiment was performed
- You should include a definition of biological versus technical replication
- The data obtained should be provided and sufficient information should be provided to indicate the number of independent biological and/or technical replicates
- If you encountered any outliers, you should describe how these were handled
- Criteria for exclusion/inclusion of data should be clearly stated
- High-throughput sequence data should be uploaded before submission, with a private link for reviewers provided (these are available from both GEO and ArrayExpress)

Please outline where this information can be found within the submission (e.g., sections or figure legends), or explain why this information doesn't apply to your submission:

We provide a description of all the data that was acquired from human subjects as well as their inclusion and exclusion criteria in Pages 16-18. We identified 4 outliers in white matter integrity measures using Tukey's method (1.5 × interquartile range), as explained in Pages 9 and 25 and in the legends of Figures 3 and 4. Our study does not include biological replicates, technical replicates or high-throughput sequence data. We collected human neuroimaging data using positron emission tomography and magnetic resonance imaging as well as clinical data.

Statistical reporting

- Statistical analysis methods should be described and justified

eLife Sciences Publications, Ltd is a limited liability non-profit non-stock corporation incorporated in the State of Delaware, USA, with company number 5030732, and is registered in the UK with company number FC030576 and branch number BR015634 at the address 1st Floor, 24 Hills Road, Cambridge CB2 1JP | August 2014

- Raw data should be presented in figures whenever informative to do so (typically when N per group is less than 10)
- For each experiment, you should identify the statistical tests used, exact values of N, definitions of center, methods of multiple test correction, and dispersion and precision measures (e.g., mean, median, SD, SEM, confidence intervals; and, for the major substantive results, a measure of effect size (e.g., Pearson's r, Cohen's d)
- Report exact p-values wherever possible alongside the summary statistics and 95% confidence intervals. These should be reported for all key questions and not only when the p-value is less than 0.05.

Please outline where this information can be found within the submission (e.g., sections or figure legends), or explain why this information doesn't apply to your submission:

The statistical analyses are described in Pages 24 and 25, where we included all the information about the tests that were used (Chi-squared, Mann-Whitney, permutation tests) and methods applied for multiple comparisons (FDR) as well as effect sizes (Spearman's Rho). We report the sample characteristics and number of subjects per group in Table 1 along with their means and SDs for clinical tests. Information about the center where data was collected can be found in Pages 16-18. We report all the exact p values for key questions in Tables 2, 3, 4 and Supplementary Tables 1, 2, regardless of their statistical significance. Raw data is presented in Table 2, Figure 3 and Figure 4. The image preprocessing analyses are described in Pages 19-23.

(For large datasets, or papers with a very large number of statistical tests, you may upload a single table file with tests, Ns, etc., with reference to sections in the manuscript.)

Group allocation

- Indicate how samples were allocated into experimental groups (in the case of clinical studies, please specify allocation to treatment method); if randomization was used, please also state if restricted randomization was applied
- Indicate if masking was used during group allocation, data collection and/or data analysis

Please outline where this information can be found within the submission (e.g., sections or figure legends), or explain why this information doesn't apply to your submission:

We included information about how subjects were assigned to different groups in Page 5. Masking was performed in the data analyses of gray matter volumes (described in Page 25), functional connectivity correlations (described in Page 8) and in the joint independent component analyses of tau and amyloid PET data (described in Page 20).

Additional data files ("source data")

- We encourage you to upload relevant additional data files, such as numerical data that are represented as a graph in a figure, or as a summary table
- Where provided, these should be in the most useful format, and they can be uploaded as "Source data" files linked to a main figure or table
- Include model definition files including the full list of parameters used
- Include code used for data analysis (e.g., R, MatLab)
- Avoid stating that data files are "available upon request"

Please indicate the figures or tables for which source data files have been provided:

eLife Sciences Publications, Ltd is a limited liability non-profit non-stock corporation incorporated in the State of Delaware, USA, with company number 5030732, and is registered in the UK with company number FC030576 and branch number BR015634 at the address 1st Floor, 24 Hills Road, Cambridge CB2 1JP | August 2014

We have uploaded the data used to create Figure 3 and Figure 4.

eLife Sciences Publications, Ltd is a limited liability non-profit non-stock corporation incorporated in the State of Delaware, USA, with company number 5030732, and is registered in the UK with company number FC030576 and branch number BR015634 at the address 1st Floor, 24 Hills Road, Cambridge CB2 1JP | August 2014
